# Network pharmacology prediction and molecular docking-based strategy to discover the potential pharmacological mechanism of Huang–Qi–Gui–Zhi–Wu–Wu decoction against deep vein thrombosis

**DOI:** 10.1186/s13018-023-03948-6

**Published:** 2023-06-30

**Authors:** Wei Fan, Shuangli Lan, Yunkang Yang, Jie Liang

**Affiliations:** 1 Department of Orthopaedics, The Affiliated Hospital of Southwest Medical University, Luzhou, China; 2 Sichuan Provincial Laboratory of Orthopaedic Engineering, Luzhou, China; 3 Department of Hepatobiliary Surgery, The Affiliated Hospital of Southwest Medical University, Luzhou, 646000 Sichuan China

**Keywords:** Huang–Qi–Gui–Zhi–Wu–Wu decoction, Deep vein thrombosis, Network pharmacology, Molecular docking, Traditional Chinese medicine

## Abstract

**Background:**

Huangqi Guizhi Wuwu decoction (HQGZWWD) has been used to treat and prevent deep vein thrombosis (DVT) in China. However, its potential mechanisms of action remain unclear. This study aimed to utilize network pharmacology and molecular docking technology to elucidate the molecular mechanisms of action of HQGZWWD in DVT.

**Methods:**

We identified the main chemical components of HQGZWWD by reviewing the literature and using a Traditional Chinese Medicine Systems Pharmacology (TCMSP) database. We used GeneCards and Online Mendelian Inheritance in Man databases to identify the targets of DVT. Herb-disease-gene-target networks using Cytascape 3.8.2 software; a protein–protein interaction (PPI) network was constructed by combining drug and disease targets on the STRING platform. Additionally, we conducted Gene Ontology (GO) and Kyoto Encyclopedia of Genes and Genomes (KEGG) enrichment analyses. Finally, molecular docking verification of active components and core protein targets was conducted.

**Results:**

A total of 64 potential targets related to DVT were identified in HQGZWWD, with 41 active components; quercetin, kaempferol, and beta-sitosterol were the most effective compounds. The PPI network analysis revealed that AKT1, IL1B, and IL6 were the most abundant proteins with the highest degree. GO analysis indicated that DVT treatment with HQGZWWD could involve the response to inorganic substances, positive regulation of phosphorylation, plasma membrane protein complexes, and signaling receptor regulator activity. KEGG analysis revealed that the signaling pathways included pathways in cancer, lipid and atherosclerosis, fluid shear stress and atherosclerosis, and the phosphatidylinositol 3-kinases/protein kinase B(PI3K-Akt) and mitogen-activated protein kinase (MAPK) signaling pathways. The molecular docking results indicated that quercetin, kaempferol, and beta-sitosterol exhibited strong binding affinities for AKT1, IL1B, and IL6.

**Conclusion:**

Our study suggests that AKT1, IL1B, and IL6 are promising targets for treating DVT with HQGZWWD. The active components of HQGZWWD likely responsible for its effectiveness against DVT are quercetin, kaempferol, and beta-sitosterol, they may inhibit platelet activation and endothelial cell apoptosis by regulating the PI3K/Akt and MAPK signaling pathways, slowing the progression of DVT.

**Supplementary Information:**

The online version contains supplementary material available at 10.1186/s13018-023-03948-6.

## Introduction

Deep vein thrombosis (DVT) is a disease in which blood in the deep veins of the limbs abnormally coagulates into clots due to damage to the venous wall and stagnation of blood flow, leading to narrowing or occlusion of the venous lumen [1, 2]. The incidence of DVT is high; for example, hospital-acquired DVT after major orthopedic surgery can reach 60% [3–5]. The danger lies in the fact that detachment of a DVT can cause fatal pulmonary embolism with a high mortality rate; it is a crucial cause of perioperative and unexpected hospital deaths [6, 7]. Low-molecular-weight heparin and vitamin K antagonists, such as warfarin and sulodexide, are routinely used in clinical practice to prevent DVT [8]; however, adverse reactions, mainly bleeding, hematoma formation, and decreased hemoglobin concentrations, have been discovered with the long-term, extensive clinical use. Furthermore, there is controversy on anticoagulants preventing DVT in patients with cerebral hemorrhage or combined blood system diseases [9]. Therefore, the effective prevention of DVT in long-term bedridden patients without causing new complications remains an urgent problem that needs to be solved.

Traditional Chinese medicine has a long history of use for the prevention and treatment of DVT [10]. Traditional Chinese medicine categorizes DVT as “pulse obstruction” and “blood stasis.” [11] Sun Simiao, a renowned Chinese physician over a thousand years ago, noted in Qian Jin Bei Ji Yao Fang that poor blood circulation is the root cause of thrombosis: “if qi and blood are stagnant, there will be pain; if the pulse is blocked, there will be swelling, and if stagnation persists for a long time, heat will arise.” HQGZWWD is composed of five traditional Chinese medicines: Huangqi (*Hedysarum multijugum maxim*, HM), Guizhi (*Cinnamomi Ramulus*, CR), Baishao (*Paeoniae Radix Alba*, PA), Shengjiang (*Zingiber officinale* *Roscoe*, ZR), and Dazao *(Jujubae Fructus*, JF) [12]. HQGZWWD is a medicinal formula that promotes blood circulation and nourishes the qi; this was reported by Zhang Zhongjing, a medical expert from the Eastern Han Dynasty (approximately 154–219 AD), in his book “Jin Gui Yao Lue” almost 2000 years ago [13]. Additionally, various medical books such as “San Yin Ji Yi Bing Zheng Fang Lun” from the Southern Song Dynasty (1174 AD), “Zheng Zhi Zhun Sheng: Lei Fang” from the Ming Dynasty (1602 AD) and “Yi Fang Ji Jie” from the Qing Dynasty recorded the effectiveness of HQGZWWD in treating DVT. Studies such as Yuebao have shown that using HQGZWWD can enhance hypercoagulability in patients who have undergone knee replacement surgery while decreasing the likelihood of developing postoperative DVT [14]. Furthermore, Zhao Zhili used HQGZWWD to treat lung cancer accompanied by DVT, significantly improving patients’ clinical symptoms and considerably shortening the acute or subacute period of DVT [15]. Despite its historical significance, our understanding of its mechanism primarily relies on the traditional Chinese medicine experience passed down through generations and clinical observations, without any scientific research regarding its molecular mechanisms.

The complexity of traditional Chinese medicine stems from the lack of adequate quantitative evidence to assess its therapeutic efficacy. Typically, traditional Chinese medicine involves intricate formulas comprising multiple herbs, with their composition and dosage often based on ancient texts and empirical knowledge rather than on contemporary scientific research [16]. Its peculiarity lies in its exclusive history within China, which has been subject to limited theoretical and fundamental investigations until recently. Consequently, many Western Scholars may not regard it as a science but rather an empirical practice lacking any theoretical foundation; these factors pose unique challenges to traditional Chinese medicine research [17].

Network pharmacology is a novel analytical approach that utilizes virtual computing and database retrieval to study the mechanisms of disease and drug action within a broader biological network. This method provides valuable insights into the pharmacological efficacy and mechanisms of the drugs [18]. The primary objective of network pharmacology research is to systematically address scientific challenges at multiple levels, which closely aligns with the fundamental concept of treating prediseases in traditional Chinese medicine, known as syndrome differentiation and treatment [19]. As an emerging research methodology, it offers a promising solution to overcome obstacles such as inadequate basic research on traditional Chinese medicine [20]. Based on this situation,We utilized network pharmacology methods to establish an herb-disease-gene-target network and constructed a protein–protein interaction (PPI) network combining drug and disease targets. We conducted a Gene Ontology (GO) analysis and Kyoto Encyclopedia of Genes and Genomes (KEGG) enrichment analysis to identify the primary signaling pathways and biological functions. Finally, molecular docking was performed to verify functional components and their major targets. In summary, our study investigates the main components of HQGZWWD that act on DVT and their molecular biological mechanisms using network pharmacology and molecular docking methods. This study provides a reference for future research in this field.

## Materials and methods

### Screening of the active components in HQGZWWD

The TCMSP platform (http://tcmspw.com/tcmsp.php) was used to identify the active components of HQGZWWD (accessed on March 15, 2023). Our selection criteria were based on the absorption, distribution, metabolism, and excretion (ADME) processes. Oral bioavailability (OB) is the amount of a drug that enters the circulation after entering the human body. However, drug-likeness (DL) refers to the degree of similarity between a compound and known drugs. OB is a representative pharmacokinetic parameter in ADME, and DL is used as a qualitative concept in drug design to estimate the molecular characteristics of drugs [21]. Specifically, we screened for components with oral bioavailability (OB) ≥ 30% and drug-like properties (DL) ≥ 0.18 to obtain the appropriate active compounds [22–25].

### Construction of drug active component target network and identification of DVT-predictive targets

We identified the active components of HQGZWWD by applying predefined thresholds (OB ≥ 30% and DL ≥ 0.18) to the TCMSP database and individually confirmed the respective targets. To standardize the target and gene symbols, we used the UniProt database (accessed on March 16, 2023) for conversion (https://www.uniprot.org/). To obtain disease targets from the databases, we searched for “deep venous thrombosis” as a keyword in the OMIM (https://omim.org/, accessed on March 16, 2023) and Gene cards databases (https://www.genecards.org/, accessed on March 16, 2023) [26, 27]. Next, we merged these targets, removed duplicates, and identified the remaining DVT targets. Using Cytascape (ver.3.8.2) network visualization software, we constructed a “herb-disease-gene-target” network of effective components and action targets. Finally, we analyzed network characteristics to elucidate the interactions between effective components and targets in herbs and diseases.

### Construction and analysis of the PPI network

The Search Tool for Retrieving Interacting Genes/Proteins (STRING) (https://string-db.org/) was used to predict protein–protein interactions (accessed on March 18, 2023). The PPI network was created by introducing overlapping targets [28]. CytoScope (ver.3.8.2) was used to construct a core PPI network.

### GO enrichment analysis and KEGG pathway analysis

To elucidate the biological functions and signaling pathways associated with DVT, we conducted GO and KEGG enrichment analyses using the Metascape database (https://metascape.org/, accessed on March 18, 2023) [29]. GO analysis identified relevant biological processes (BP), cellular components (CC), and molecular functions (MF). Additionally, KEGG enrichment analysis enabled us to identify significant signaling pathways involved in these biological processes. The *P*-value cutoff was established as *P*<0.01, where a lesser *P*-value signifies a higher likelihood of the current result being an authentic enrichment outcome rather than a random occurrence. The unit employed in this study was -log _10_(*P*-value), and as this value increased, the reliability of the enrichment results increased.

### Molecular docking

Molecular docking is a theoretical simulation method used in drug design to predict the binding modes and affinities of molecules by studying their interactions with receptors and ligands [30]. AutoDock Tools is a simulation software that facilitates the study of the interactions between biomolecules and small-molecule complexes; this enables researchers to accurately comprehend how protein targets interact with small-molecule compounds by simulating the ligand-receptor recognition [31]. In this study, we employed molecular docking to investigate whether the core components of HQGZWWD, identified through network pharmacology, could bind to core proteins. We selected the top three compounds based on degree value from the “herb-disease-gene-target” network's core components of HQGZWWD and chose the top three proteins based on degree value from the PPI network's core targets. The corresponding 3D structure files for proteins and small molecule compounds were downloaded from the RCSB database (https://www.rcsb.org/, accessed on March 19, 2023) and TCMSP database [32], followed by dehydration and hydrogenation before importing them into Autodock Tools (ver.1.5.6). The small molecule compound was then docked with the protein as a receptor using a molecular docking method to select the most suitable conformation. Finally, to generate a binding mode diagram, we imported the docked protein and small molecule files prepared using AutoDock Tools into PyMOL (ver.1.8.x). Using the 'find-polar contacts-to any atom' function, we can automatically identify hydrogen bonds or other interactions [33].

## Results

### Extraction of the active components in HQGZWWD

According to the screening criteria, OB ≥ 30% and DL ≥ 0.18 were set in the TCMSP database for screening; the results showed that 41 active components of HQGZWWD were obtained, as shown in Additional file 1.

### Identification of core targets and construction of herb-disease-gene-target network

**Fig. 1:**
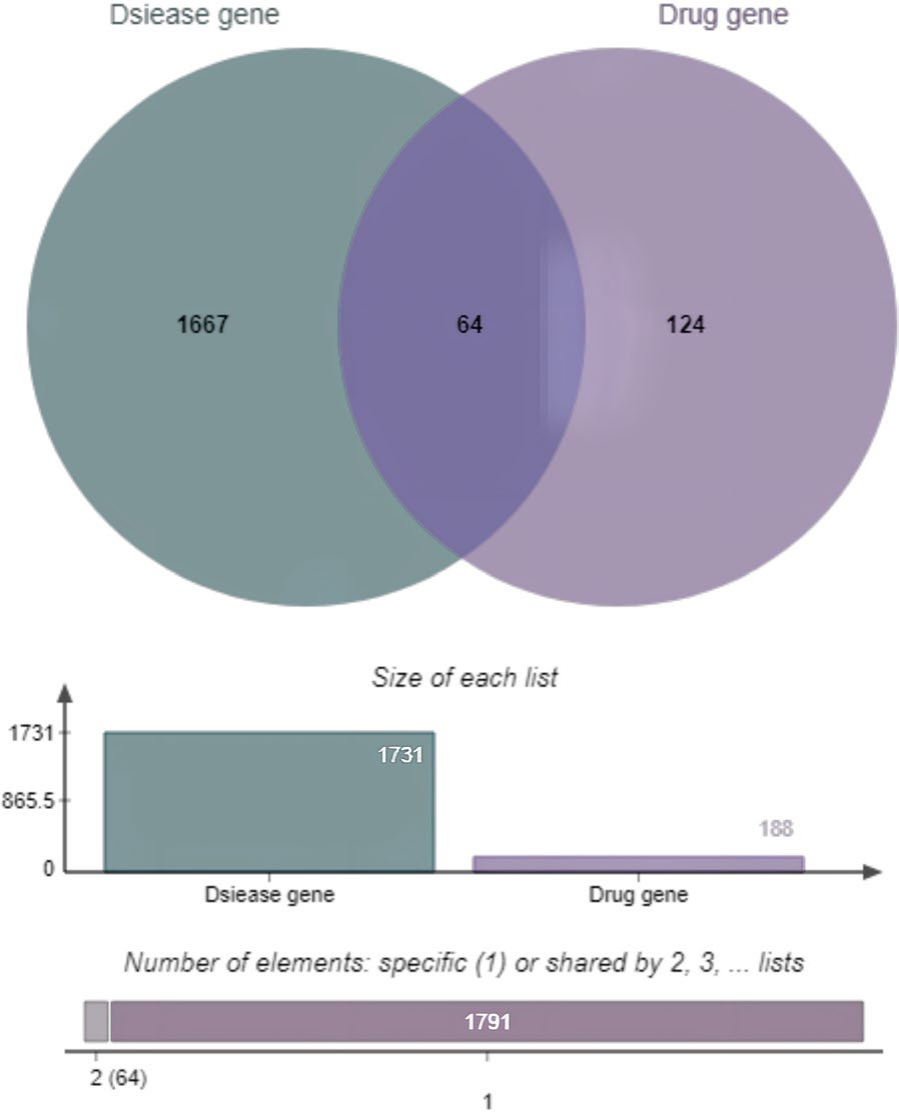
Venn diagram showing the overlapping target genes for HQGZWWD against DVT. HQGZWWD, Huang-Qi-Gui-Zhi-Wu-Wu Decoction; DVT, Deep vein thrombosis

**Fig. 2:**
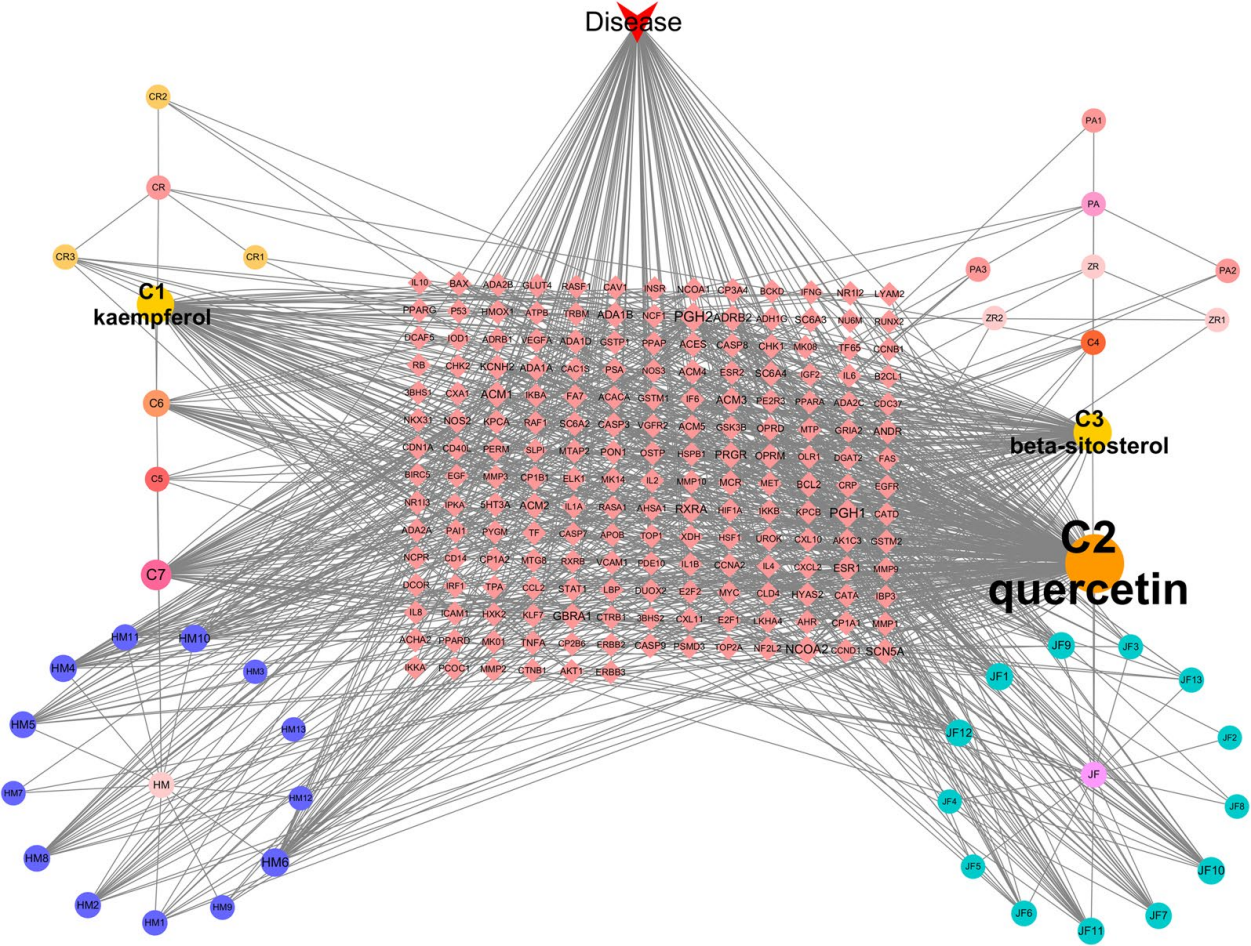
Herb-disease-gene-target network of HQGZWWD against DVT. The larger the font size, the more important its role in the compound. *HQGZWWD* Huang-Qi-Gui-Zhi-Wu-Wu Decoction, *DVT* Deep vein thrombosis, *HM* Hedysarum multijugum Maxim, *CR* Cinnamomi Ramulus, *PA* Paeoniae Radix Alba, *ZR* Zingiber officinale Roscoe, *JF* Jujubae Fructus. (C1)kaempferol, common components of HM and PA, (C2)quercetin, common components of HM and JF, (C3)beta-sitosterol, common components of CR, PA, ZR, and JF, (C4)sitosterol, common components of CR and PA, (C5)mairin, common components of HM, PA, and JF, (C6)( +)-catechin, common components of CR, PA, and JF, (C7)stigmasterol, common components of ZR and JF

We searched the OMIM and GeneCards databases for potential targets related to DVT, removed duplicate targets, and identified 1731 potential targets. We then sorted the results from TCMSP, removed duplicate sites, matched the predicted drug targets with the corresponding disease targets, and obtained 64 core targets (Fig. 1). To further observe the compound-target interactions, we constructed a "herb-disease-gene-target" network (Fig. 2). The degree refers to the number of nodes that interact with a node, and its size represents its core degree. The higher the degree value, the more important this node is. Our findings indicate that three compounds in HQGZWWD are highly correlated with DVT: quercetin (C2; degree = 246), beta-sitosterol (C3; degree = 100), and kaempferol (C1; degree = 94).

### Construction and analysis of the PPI network

**Fig. 3:**
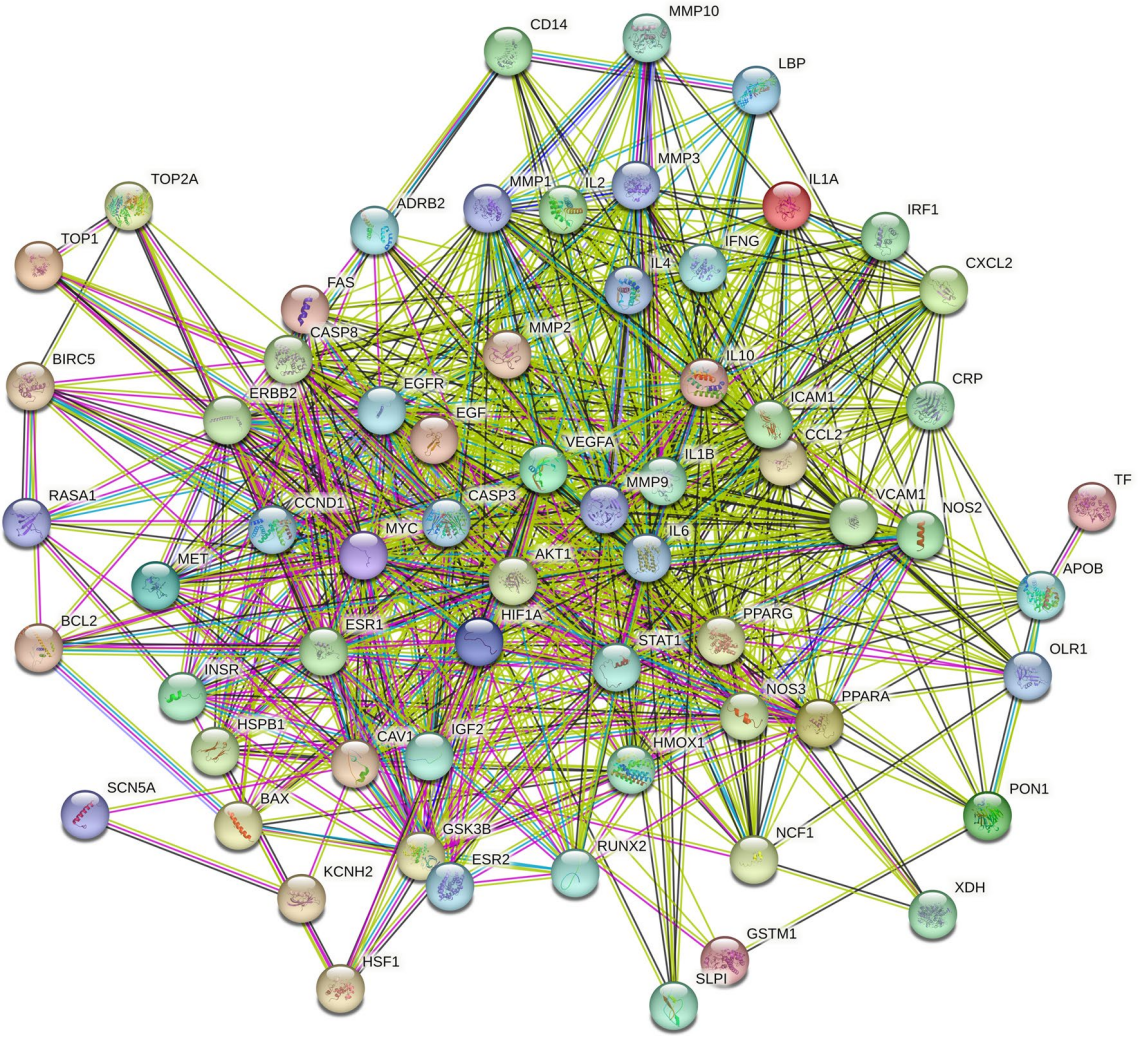
PPI network. Empty nodes represent proteins of unknown 3D structures; filled nodes represent some 3D structures that are known or predicted. Edges represent protein–protein associations: the light blue edges represent from curated databases; the fuchsia edges represent experimentally determined; the green edges represent gene neighborhood; the red edges represent gene fusions; the dark blue edges represent gene co-occurrence; the light green edges represent text mining; the black edges represent co-expression; the light purple edges represent protein homology

**Fig. 4:**
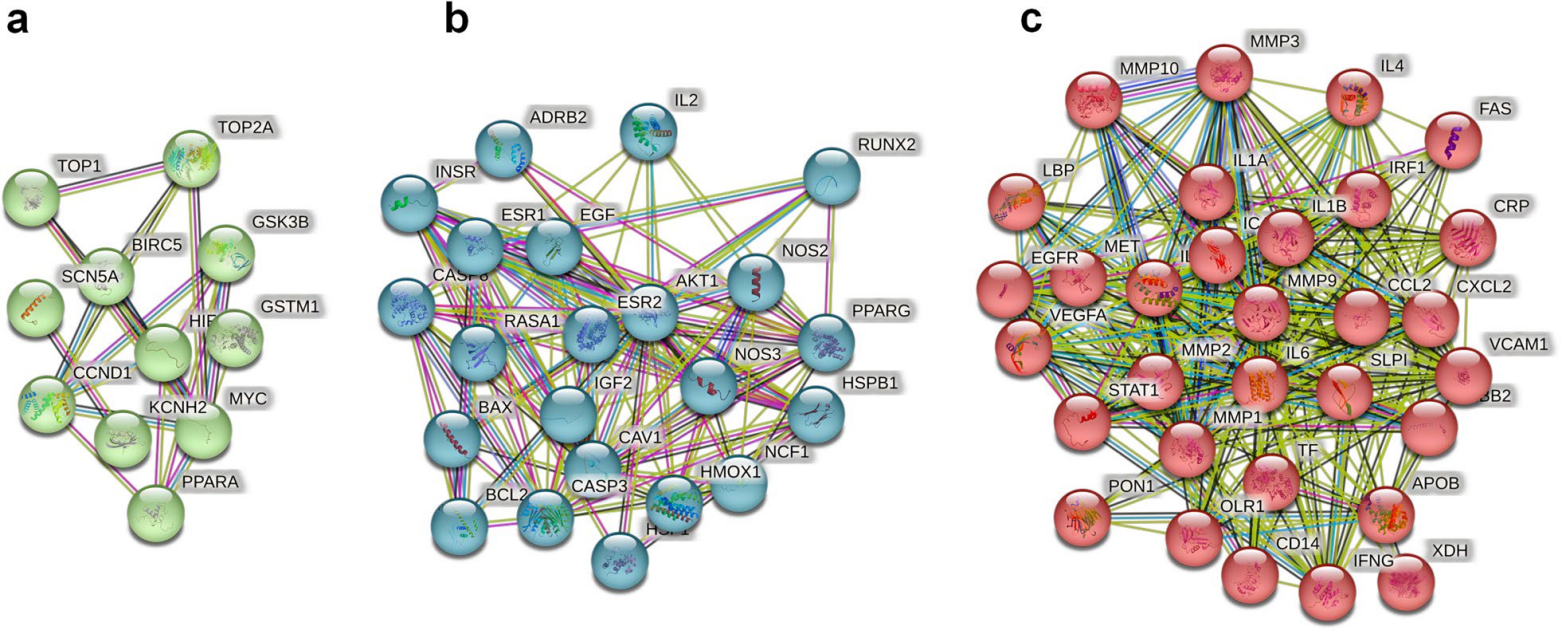
Central gene cluster identified in the PPI network of HQGZWWD-DVT based on MCODE analysis. **a**–**c** Was cluster 1, 2, and 3, respectively

**Fig. 5:**
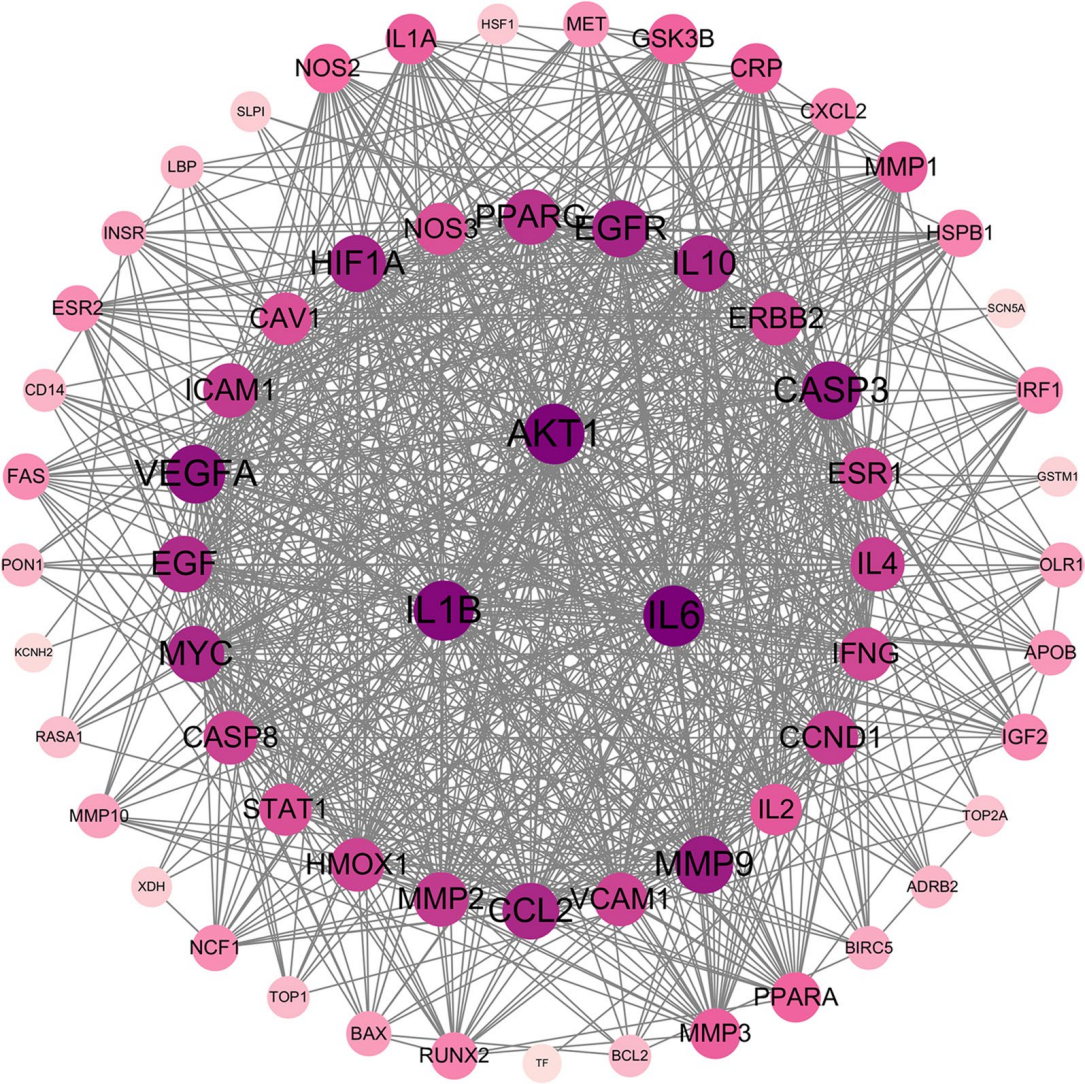
PPI network diagram processed by Cytascape. The color of the target point changes gradually according to the degree value. The higher the degree value, the larger the circle. As the degree value changes, the color changes from light purple to deep purple

The core overlapping diseases and drug component targets were imported into the STRING database to generate a PPI network diagram (Fig. 3). Molecular complex detection (MCODE) is a novel graph-theoretic clustering algorithm that calculates the information of each node in a PPI network graph using a k-means clustering algorithm to detect dense connection regions in large protein-protein interactions. Proteins within these regions may have similar structures or functions, providing a reference and guidance for further research on protein-disease interactions [34]. Target information from this network was further analyzed using MCODE, which revealed three central gene clusters; these clusters suggested that the proteins within them were closely connected and may share common functions or expression patterns (Fig. 4). Key target proteins were identified using the Cytoscape software, with higher degree values indicating stronger interactions with other targets. This analysis highlighted the potential central targets for HQGZWWD in DVT treatment, including AKT1 (degree = 51), IL6 (degree = 52), and IL1B (degree = 50) (Fig. 5).

### GO enrichment analysis and KEGG pathway analysis

**Fig. 6:**
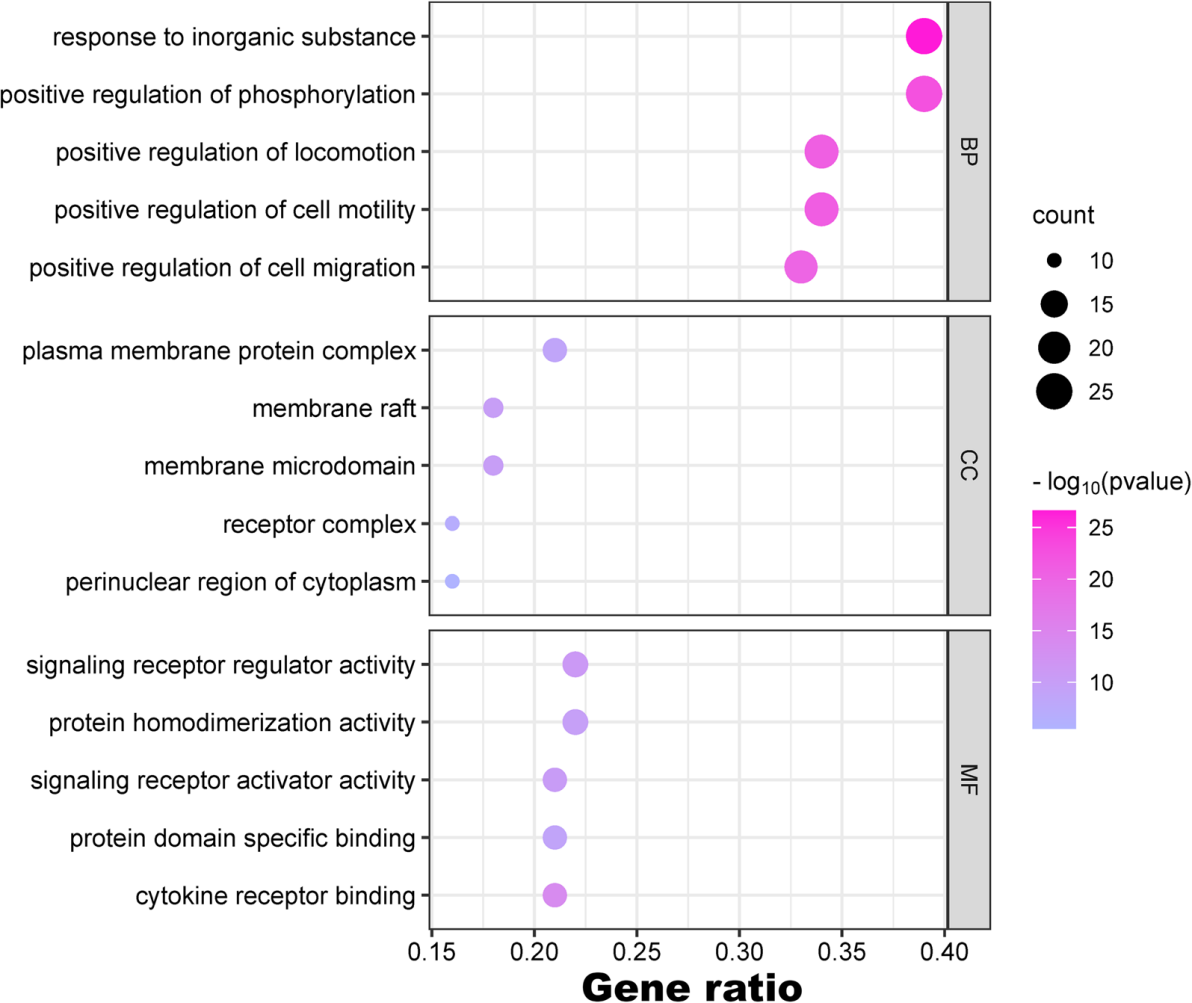
GO analysis of key target genes. The top 5 items of biological function are listed on the vertical axis, including GOMF, GOCC, and GOBP, the horizontal axis in the figure represents the gene ratio. *GO* Gene ontology, *BP* biological process, *CC* cell composition

**Fig. 7:**
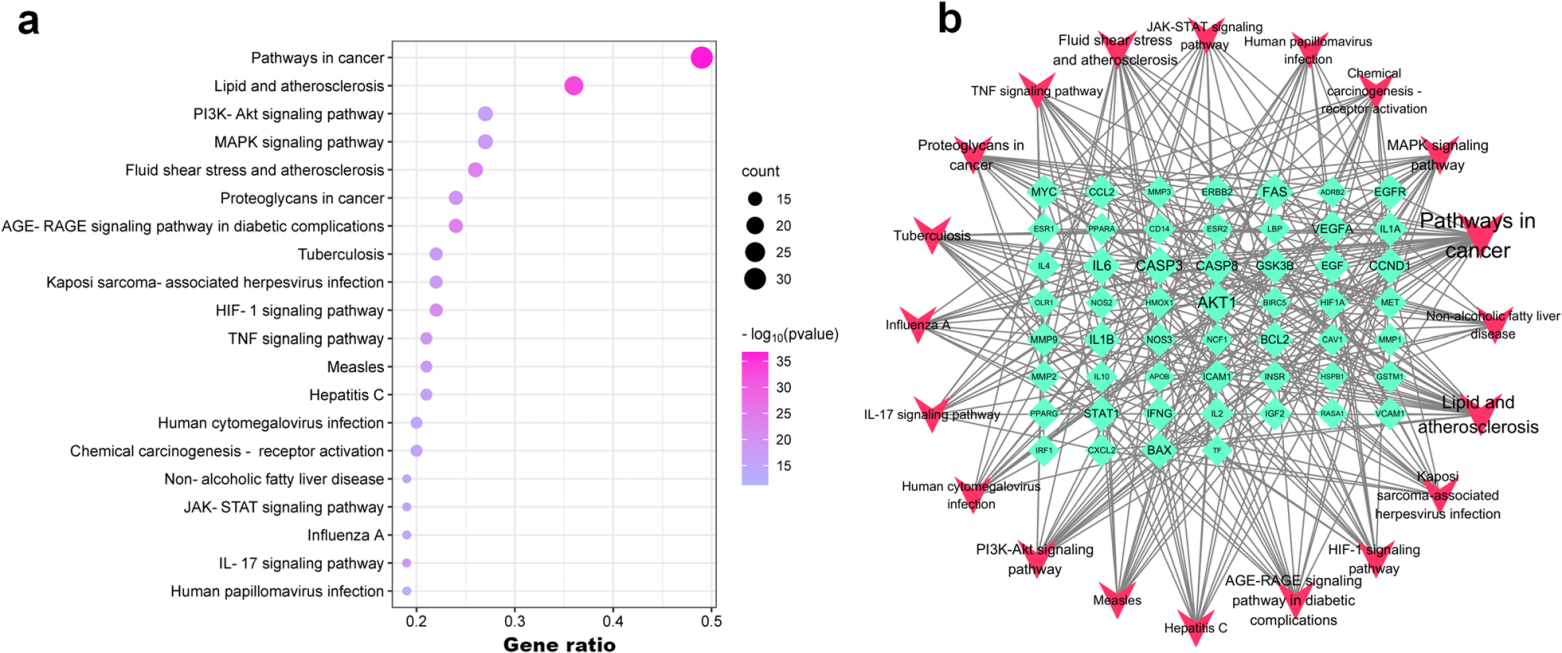
**a** KEGG analysis of key target genes, **b** Network of top 20 pathways. Red diamond represents gene, and green triangle represents pathway. The size of the nodes represents the value of the degree. The horizontal axis in the figure represents the gene ratio. *KEGG* Kyoto Encyclopedia of Gene and Genome

GO and KEGG enrichment analyses were conducted to better understand the potential pathways and biological functions of HQGZWWD in DVT. Figure 6 displays the top five results of the GO analysis, which revealed that HQGZWWD primarily affected the response to inorganic substances (BP), positive regulation of phosphorylation (BP), plasma membrane protein complex (CC), and signaling receptor regulator activity (MF). Additionally, our KEGG analysis identified several signaling pathways involved in this treatment approach, including pathways in cancer, lipid and atherosclerosis, fluid shear stress and atherosclerosis, the PI3K-Akt pathway, and the MAPK signaling pathway (Fig. 7a, b).

### Molecular docking

**Fig. 8:**
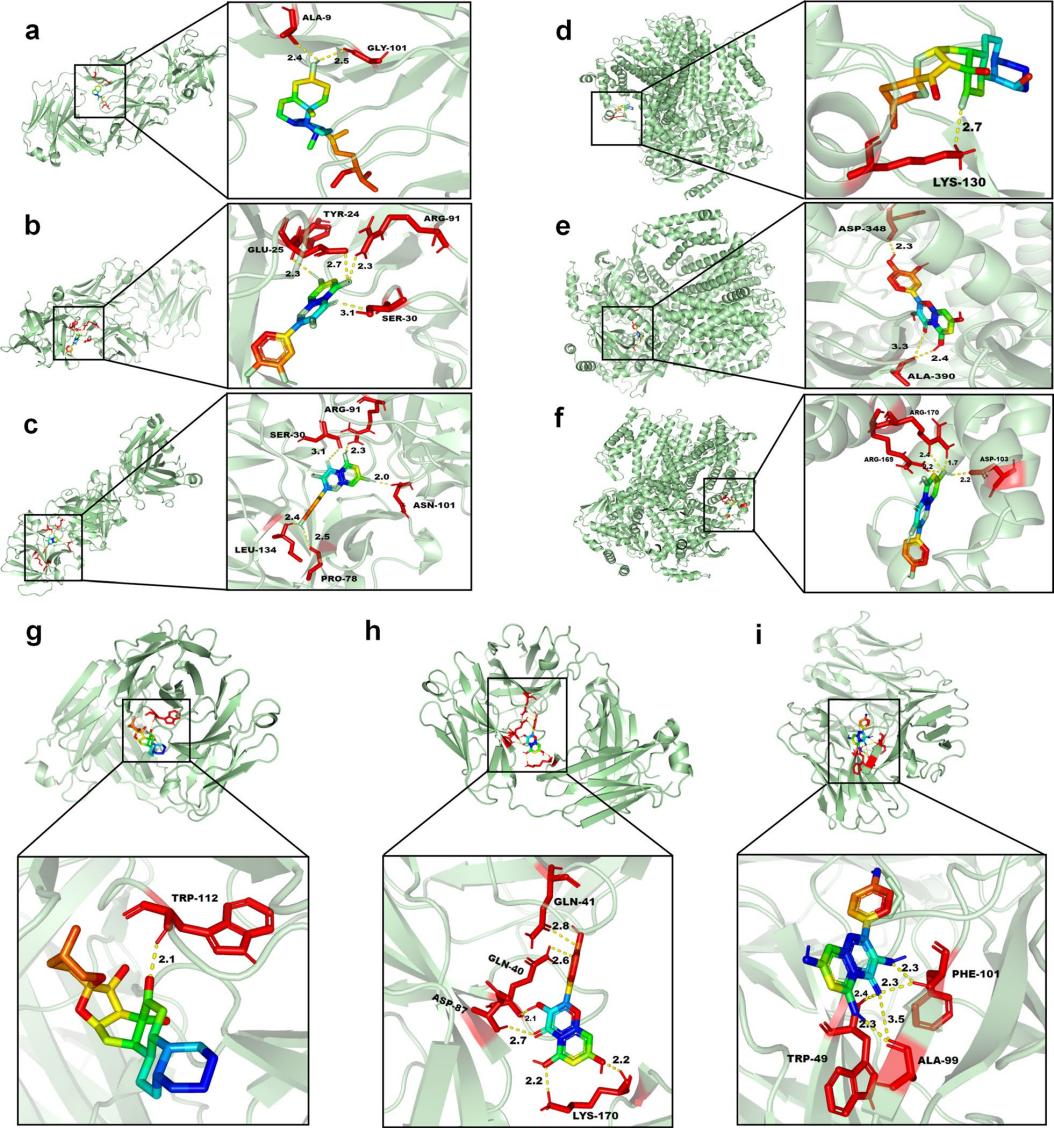
Molecular docking results of main chemical components of HQGZWWD and core proteins in PPI network **a** IL1B-beta-sitosterol, **b** IL1B-quercetin, **c** IL1B-kaempferol, **d** AKT1-beta-sitosterol, **e** AKT1-quercetin, **f** AKT1-kaempferol, **g** IL6-beta-sitosterol, **h** IL6-quercetin, **i** IL6-kaempferol

We employed network pharmacology and PPI analyses to determine the top three compounds, quercetin, beta-sitosterol, and kaempferol, for docking with the top three proteins, AKT1, IL6, and IL1B. The best docking image of the receptor-ligand complex is presented in Fig. 8.

Our results indicate that beta-sitosterol can form hydrogen bonds with ALA-9 and GLY-101 through IL-1B protein (Fig. 8a), while quercetin can do so through GLU-25, TYR-24, ARG-91, and SER-30 (Fig. 8b), and kaempferol through SER-30, ARG-91, ASN-101, LEU-134, and PRO-78 (Fig. 8c). Similarly, for AKT1, beta-sitosterol formed hydrogen bonds via LYS-130 (Fig. 8d) and quercetin via ASP-348 and ALA-390 (Fig. 8e), while kaempferol formed hydrogen bonds via ARG-169/170 and ASP-103 (Fig. 8f). Finally, for the IL6 protein: beta-sitosterol forms hydrogen bonds via TRP-112 (Fig. 8g); quercetin does so through GLN-41/40, ASP-87, and LYS-170 (Fig. 8h), while kaempferol forms bonds between PHE-101/TR-P-49 and ALA-99 (Fig. 8i).

The tighter the binding between the ligands and receptors, the smaller the binding energy. Notably, binding energy < -5 kcal/mol indicates good affinity and binding activity between the receptor and ligand [35, 36]. Our molecular docking results showed that all three selected compounds (quercetin, beta-sitosterol, and kaempferol) had high affinities for the three core targets (AKT1, IL6, and IL1B), as their binding energies to proteins were < -5 kcal/mol (Table 1); this suggests that these compounds may play crucial roles in the treatment of DVT.

**Table 1:** Binding energy of molecular docking

Target	Target (PDB ID)	Target structure	Compound	Binding energy (kcal/mol)
IL1B	7Z4T		Beta-sitosterol (Fig. 8a)	− 8.0
			Quercetin (Fig. 8b)	− 8.1
			Kaempferol (Fig. 8c)	− 8.2
AKT1	7FCV		Beta-sitosterol (Fig. 8d)	− 9.7
			Quercetin (Fig. 8e)	− 7.8
			Kaempferol (Fig. 8f)	− 8.3
IL6	7PHS		Beta-sitosterol (Fig. 8g)	− 8.7
			Quercetin (Fig. 8h)	− 7.8
			Kaempferol (Fig. 8i)	− 7.5

## Discussion

This study investigated the mechanism by which HQGZWWD treats DVT. By constructing and analyzing databases and network diagrams of traditional Chinese medicine prescriptions, we identified quercetin, beta-sitosterol, and kaempferol as highly correlated with DVT. Previous studies demonstrated that quercetin and its derivatives possess anticoagulant, antiplatelet, and antifibrinolytic activities [37, 38], effectively preventing pulmonary thromboembolism [39]. Furthermore, onion extracts rich in quercetin regulate MAPK under coagulation stimulation to prolong thrombosis time [40], consistent with our KEGG enrichment analysis results. Kaempferol and quercetin are flavonoids that inhibit prothrombin activity while regulating fibrinogen-thrombin interactions for antithrombotic formation in vivo and in vitro [41]. In 2018, researchers reported that plant-derived beta-sitosterol exhibits antithrombotic properties, such as in vivo anticoagulant effects and thromboprophylaxis [42]. These findings suggest that these components play an essential role in the treatment of DVT using HQGZWWD. However, research on the mechanisms of action of beta-sitosterol and kaempferol in thrombosis is still at an early stage and warrants further exploration.

The results of the PPI network analysis suggested that AKT1, IL6, and IL1B could be potential core targets for treating DVT with HQGZWWD; IL-1B and IL-6 are proinflammatory cytokines with a wide range of biological activities [43]. Initially, it was believed that IL6 did not play a role in venous thrombosis; however, as more research was conducted on its function, this statement was discarded [44]. Studies have revealed that patients with DVT have elevated levels of IL-6, and in vivo and in vitro studies have confirmed its involvement in DVT formation [45, 46]. Similar to psoriasis, studies have shown that IL-6 is involved in regulating inflammation-related thrombosis [47]. Regarding IL-1B, research has indicated increased body levels during acute pulmonary embolism, which can be reversed by atorvastatin treatment [48]. Currently, most studies on cytokines focus on their effects on atherosclerosis and cardiovascular disease [49–51]; however, research on their direct involvement in venous thrombosis started late. Further exploration is needed to determine the specific mechanism underlying these findings, which were significant in our study.

We conducted GO and KEGG analyses to investigate the mechanism of action of HQGZWWD in DVT. The results of the GO analysis showed that HQGZWWD treatment primarily involved responses to inorganic tolerance, positive regulation of phosphorylation, plasma membrane protein complex, and signaling receiver regulator activity. KEGG analysis revealed that multiple signaling pathways are involved in HQGZWWD treatment of DVT, including pathways in cancer, lipid and atherosclerosis, fluid shear stress, atherosclerosis, PI3K-Akt, and MAPK signaling pathways. The involvement of quercetin in cancer pathogenesis may be the main reason for its enrichment in cancer [52–54]. A previous study showed that the lack of protein tyrosine phosphatase non-receptor type 22 (PTPN22) significantly accelerates thrombus formation. Further research has shown that in platelets with PTPN22 defects, the phosphorylation of phosphodiesterase 5A (PDE5A) increases, and the cyclic guanosine monophosphate levels and vasodilator-stimulated phosphoprotein phosphorylation decrease [55]. Other studies have demonstrated that the platelet phenotypic response caused by Glycoprotein VI (GPVI) activation is linked to changes in protein kinase substrate phosphorylation. To investigate how adenosine diphosphate (ADP) secretion and thromboxane generation feedback affect GPVI activation and signal transduction, proteomic analysis was conducted; the results revealed significant alterations in the phosphorylation levels following ADP secretion, thromboxane feedback, and collagen-related peptide (CRP-XL) stimulation. The significantly altered proteins were primarily involved in biological processes such as vesicle-mediated transport and cell skeleton reconstruction, which cannot be separated from the role of quality model protein complexes and signal regulation receptor activity [56]. These identified biological processes, cellular components, and molecular functions aligned with our GO analysis results: response to inorganic tolerance, positive regulation of phosphorylation, plasma membrane protein complex, and signaling receiver regulator activity; therefore, it is worth investigating whether HQGZWWD exerts antagonistic effects on DVT formation via these biological processes. Our confidence was enhanced by the preliminary prediction results of the network pharmacology.

Although atherosclerosis and venous thrombosis were once considered distinct pathological conditions, recent studies have challenged this notion [57]. A study published in the NEJM suggested that atherosclerosis may contribute to the development of venous thrombosis [58]. Furthermore, Hong et al. discovered that patients with idiopathic DVT had significantly higher rates of coronary artery calcification than controls [59]. Another study reported that patients with acute DVT had a high incidence of acute myocardial infarction or ischemic stroke-related death during long-term follow-up [60]. These findings suggest a potential synergistic relationship between atherosclerosis and venous thrombosis. For instance, some studies have demonstrated that atherosclerosis can affect the adhesion of thrombotic substances to the peripheral venous system [61]. Specifically, atherosclerosis primarily affects the arterial tree system and is caused by an imbalance in lipid metabolism and maladaptive immune responses, resulting in chronic inflammation of the blood vessel walls [62–64]. Furthermore, chronic inflammatory diseases that cause changes in blood vessel walls are associated with a higher risk of DVT [65, 66]. Shared risk factors such as inflammation, hypercoagulability, and endothelial injury increase the likelihood of chronic inflammation induced by processes related to atherosclerosis, affecting the development of DVT and adhesion of DVT clots within veins [67, 68]. Traditional Chinese medicine treatment methods have shown that HQGZWWD is effective against coronary heart disease caused by arteriosclerosis, cerebral infarction, and peripheral vascular disease [69, 70]. Furthermore, literature search has revealed quercetin, a compound in HQGZWWD, can combat the progression of arteriosclerotic plaques through multiple mechanisms, such as regulating oxidized low-density lipoprotein-induced endothelial cell senescence [71] and modulating the autophagy of macrophages [72]. Since HQGZWWD has a positive therapeutic effect on arteriosclerosis, which is known to have a mutually promoting effect on DVT, it may be worthwhile to investigate whether HQGZWWD can simultaneously inhibit the progression of both conditions; this aligns with one of the objectives of network pharmacology, drug repositioning, and drug development.

The PI3K/Akt signaling pathway is a crucial regulatory pathway playing a significant role in pathophysiological processes such as cell growth, differentiation, and proliferation [73]. Recently, researchers have focused on its correlation with endothelial cell mobilization [74], differentiation [75, 76], and apoptosis [77]. Studies have suggested that this pathway regulates vascular endothelial growth factor (VEGF) secretion and controls apoptosis in blood vessels and vascular endothelial cells [78]. Additionally, microRNA-126 has been found to inhibit the PI3K/Akt signaling pathway to prevent vascular endothelial cell apoptosis for therapeutic effects in DVT treatment [79]. Furthermore, FXII activates PI3K/AKT signaling and promotes DVT progression by inducing inflammatory reactions [80]. However, kaempferol, the main component identified in this study, significantly reduced the phosphorylation of PI3K/AKT during the collagen/adrenaline-stimulated platelet activation tests. This delay ultimately resulted in a 34.6% reduction in clotting time. Additionally, animal experiments demonstrated the thromboprotective effect of resveratrol in collagen/adrenaline- and thrombin-induced acute thrombotic embolism models and FeCl3-induced carotid artery thrombosis models [41]. The target we selected was enriched in the PI3K/Akt signaling pathway, and kaempferol was one of the main components screened, which validated the presence of AKT1 in the PPI network analysis and suggested promising directions for future research. HQGZWWD can potentially inhibit endothelial cell apoptosis by activating the PI3K/Akt pathway, which could potentially slow the progression of DVT. Kaempferol, the primary constituent of HQGZWWD, may play a crucial role in this mechanism; however, additional cellular and animal experiments are necessary to further investigate this mechanism.

The MAPK signaling pathway is a crucial component of the eukaryotic signal transduction network; it plays a key role in regulating cell proliferation, differentiation, apoptosis, and stress responses under normal and pathological conditions [81, 82]. Studies have demonstrated that a sustained increase in reactive oxygen species can activate inflammatory reactions by activating MAPK signaling pathways. This activation leads to apoptosis of vascular endothelial cells and induces thrombosis [83]. Furthermore, studies have shown that ginsenoside-Rp3 can regulate MAPK signaling pathways to inhibit platelet activation and thrombosis [84]. These findings support our hypothesis that HQGZWWD may delay DVT progression by modulating the MAPK signaling pathway. Previous research has indicated that the activation of Raf1 in the MAPK pathway can regulate thromboxane production and platelet thrombosis [85]. However, quercetin, the primary compound in HQGZWWD, has been shown to inhibit IL-1 and chemokine production via the MAPK signaling pathway [86]. IL-1-induced inflammatory factors can activate proinflammatory thrombogenic processes in vascular endothelial cells, stimulate angiogenic mediator production, and promote thrombosis [87, 88]. Therefore, investigating whether HQGZWWD and its main components inhibit thrombus formation by regulating these processes is essential; the findings of this study reveal its potential.

Molecular docking experiments revealed that quercetin, beta sitosterol, and kaempferol, the main compounds obtained from HQGZWWD, have binding energies with core target proteins AKT1, TP53, and TNF of < -5 kcal/mol (Table 1). This indicated that these three small molecule compounds may bind to these proteins and regulate signal transduction; they play vital roles in the treatment of DVT. However, the therapeutic effects and molecular mechanisms of these agents active in DVT require further investigation; we believe they possess significant potential for the clinical prevention and treatment of DVT.

This study had some limitations. Although we used modern bioinformatics methods to explore the role of HQGZWWD in treating DVT through network pharmacology and molecular docking, there is still a lack of molecular dynamics(MD) simulations to supplement molecular docking in determining the dynamic stability of receptor ligands [89, 90]. Additionally, this study relied solely on conventional computational analysis without utilizing more comprehensive omics datasets for mining targets. Although quercetin, sitosterol, and kaempferol have been identified as the three most important bioactive components for treating DVT, they do not represent all the compounds in HQGZWWD. Therefore, further research involving MD simulations, deeper omics data mining, and molecular biology experiments are necessary to validate our findings.

## Conclusions

In summary, we adopted a new approach that combined network pharmacology and molecular docking to predict AKT1, IL1B, and IL6 as the most likely targets of HQGZWWD for treating DVT. Quercetin, kaempferol, and beta-sitosterol are the main active components of HQGZWWD; they may inhibit platelet activation and endothelial cell apoptosis by regulating the PI3K/Akt and MAPK signaling pathways, slowing the progression of DVT. Additionally, our results offer new insights into the pathogenesis of DVT and suggest potential avenues for developing innovative treatment strategies. The initial findings from network pharmacology have increased our confidence. However, further clinical experiments and in-depth basic research are necessary to fully understand the therapeutic effects and molecular mechanisms of traditional Chinese medicine on DVT; this will be the next area of our research.

## Supplementary Information

**Additional file: 1.** Effective compounds of Huang-Qi-Gui-Zhi-Wu-Wu Decoction.

## Data Availability

The data used and/or analyzed during the current study are available from the corresponding author upon reasonable request. The datascxsets generated and/or analysed during the current study are available in the followed database: TCMSP database (http://tcmspw.com/tcmsp.php). UniProt database (https://www.uniprot.org/). OMIM database (https://omim.org/). Gene cards database (https://www.genecards.org/). STRING database (https://string-db.org/). Metascape database (https://metascape.org/). RCSB database (https://www.rcsb.org/).

## Abbreviations

HQGZWWD: Huang–Qi–Gui–Zhi–Wu–Wu decoction
DVT: Deep vein thrombosis
GO: Gene ontology
BP: Biological process
CC: Cell composition
KEGG: Kyoto encyclopedia of gene and genome
VEGF: Vascular endothelial growth factor
OB: Oral bioavailability
DL: Drug-likeness
