# Supplementary material for: Network pharmacology prediction and molecular docking-based strategy to discover the potential pharmacological mechanism of Huang–Qi–Gui–Zhi–Wu–Wu decoction against deep vein thrombosis
Source: J Orthop Surg Res. 2023 Jun 30;18:475. doi: 10.1186/s13018-023-03948-6 (PMC10314554; doi:10.1186/s13018-023-03948-6)
Supplement: Supplementary file 1 — Additional file: 1. Effective compounds of Huang-Qi-Gui-Zhi-Wu-Wu Decoction. [file 13018_2023_3948_MOESM1_ESM.docx]

Supplementary material 1.Effective compounds of Huang-Qi-Gui-Zhi-Wu-Wu Decoction

| Name | Mol ID | Mol name | OB (%) | DL |
| --- | --- | --- | --- | --- |
| HM1 | MOL000239 | Jaranol | 50.83 | 0.29 |
| HM2 | MOL000296 | hederagenin | 36.91 | 0.75 |
| HM3 | MOL000033 | (3S,8S,9S,10R,13R,14S,17R)-10,13-dimethyl-17-[(2R,5S)-5-propan-2-yloctan-2-yl]-2,3,4,7,8,9,11,12,14,15,16,17-dodecahydro-1H-cyclopenta[a]phenanthren-3-ol | 36.23 | 0.78 |
| HM4 | MOL000354 | isorhamnetin | 49.6 | 0.31 |
| HM5 | MOL000371 | 3,9-di-O-methylnissolin | 53.74 | 0.48 |
| HM6 | MOL000378 | 7-O-methylisomucronulatol | 74.69 | 0.3 |
| HM7 | MOL000379 | 9,10-dimethoxypterocarpan-3-O-β-D-glucoside | 36.74 | 0.92 |
| HM8 | MOL000380 | (6aR,11aR)-9,10-dimethoxy-6a,11a-dihydro-6H-benzofurano[3,2-c]chromen-3-ol | 64.26 | 0.42 |
| HM9 | MOL000387 | Bifendate | 31.1 | 0.67 |
| HM10 | MOL000392 | formononetin | 69.67 | 0.21 |
| HM11 | MOL000417 | Calycosin | 47.75 | 0.24 |
| HM12 | MOL000433 | FA | 68.96 | 0.71 |
| HM13 | MOL000442 | 1,7-Dihydroxy-3,9-dimethoxy pterocarpene | 39.05 | 0.48 |
| CR1 | MOL001736 | (-)-taxifolin | 60.51 | 0.27 |
| CR2 | MOL000073 | ent-Epicatechin | 48.96 | 0.24 |
| CR3 | MOL004576 | taxifolin | 57.84 | 0.27 |
| PA1 | MOL001918 | paeoniflorgenone | 87.59 | 0.37 |
| PA2 | MOL001919 | (3S,5R,8R,9R,10S,14S)-3,17-dihydroxy-4,4,8,10,14-pentamethyl-2,3,5,6,7,9-hexahydro-1H-cyclopenta[a]phenanthrene-15,16-dione | 43.56 | 0.53 |
| PA3 | MOL001924 | paeoniflorin | 53.87 | 0.79 |
| ZR1 | MOL006129 | 6-methylgingediacetate2 | 48.73 | 0.32 |
| ZR2 | MOL001771 | poriferast-5-en-3beta-ol | 36.91 | 0.75 |
| JF1 | MOL012921 | stepharine | 31.55 | 0.33 |
| JF2 | MOL012946 | zizyphus saponin I_qt | 32.69 | 0.62 |
| JF3 | MOL012976 | coumestrol | 32.49 | 0.34 |
| JF4 | MOL012986 | Jujubasaponin V_qt | 36.99 | 0.63 |
| JF5 | MOL012992 | Mauritine D | 89.13 | 0.45 |
| JF6 | MOL001454 | berberine | 36.86 | 0.78 |
| JF7 | MOL001522 | (S)-Coclaurine | 42.35 | 0.24 |
| JF8 | MOL004350 | Ruvoside_qt | 36.12 | 0.76 |
| JF9 | MOL000627 | Stepholidine | 33.11 | 0.54 |
| JF10 | MOL007213 | Nuciferin | 34.43 | 0.4 |
| JF11 | MOL000787 | Fumarine | 59.26 | 0.83 |
| JF12 | MOL002773 | beta-carotene | 37.18 | 0.58 |
| JF13 | MOL000096 | (-)-catechin | 49.68 | 0.24 |
| C1 | MOL000422 | kaempferol | 41.88 | 0.24 |
| C2 | MOL000098 | quercetin | 46.43 | 0.28 |
| C3 | MOL000358 | beta-sitosterol | 36.91 | 0.75 |
| C4 | MOL000359 | sitosterol | 36.91 | 0.75 |
| C5 | MOL000211 | Mairin | 55.38 | 0.78 |
| C6 | MOL000492 | (+)-catechin | 54.83 | 0.24 |
| C7 | MOL000449 | Stigmasterol | 43.83 | 0.76 |

Note：(Mol) molecular, (OB ) oral bioavailability, (DL) drug-likeness,(HM) Hedysarum multijugum Maxim, (CR)Cinnamomi Ramulus, (PA)Paeoniae Radix Alba, (ZR)Zingiber officinale Roscoe, (JF)Jujubae Fructus, (C1)kaempferol, common components of HM and PA，(C2)quercetin, common components of HM and JF,

(C3)beta-sitosterol, common components of CR, PA, ZR and JF, (C4)sitosterol ,common components of CR and PA，(C5)Mairin，common components of HM，PA and JF，(C6)(+)-catechin，common components of CR，PA and JF ，(C7)Stigmasterol，common components of ZR and JF.
